# Akt isoforms in the immune system

**DOI:** 10.3389/fimmu.2022.990874

**Published:** 2022-08-23

**Authors:** Mireia Guerau-de-Arellano, Zayda L. Piedra-Quintero, Philip N. Tsichlis

**Affiliations:** ^1^ School of Health and Rehabilitation Sciences, Division of Medical Laboratory Science, College of Medicine, Wexner Medical Center, The Ohio State University, Columbus, OH, United States; ^2^ Institute for Behavioral Medicine Research, The Ohio State University, Columbus, OH, United States; ^3^ Department of Microbial Infection and Immunity, The Ohio State University, Columbus, OH, United States; ^4^ Department of Neuroscience, The Ohio State University, Columbus, OH, United States; ^5^ The Ohio State University Comprehensive Cancer Center, The Ohio State University, Columbus, OH, United States; ^6^ Department of Cancer Biology and Genetics, The Ohio State University, Columbus, OH, United States

**Keywords:** Akt, Akt1, Akt2, Akt3, immune cells

## Abstract

Akt is a PI3K-activated serine-threonine kinase that exists in three distinct isoforms. Akt’s expression in most immune cells, either at baseline or upon activation, reflects its importance in the immune system. While Akt is most highly expressed in innate immune cells, it plays crucial roles in both innate and adaptive immune cell development and/or effector functions. In this review, we explore what’s known about the role of Akt in innate and adaptive immune cells. Wherever possible, we discuss the overlapping and distinct role of the three Akt isoforms, namely Akt1, Akt2, and Akt3, in immune cells.

## Introduction

Akt, also known as Protein kinase B (PKB), is a serine-threonine kinase with important roles in proliferation, metabolism, cell differentiation, survival, etc (1, 2). There are three distinct Akt isoforms encoded by three separate genes, namely Akt1/PKBα, Akt2/PKBβ and Akt3/PKBγ. Akt activation occurs as a consequence of signals that result from phosphatidyl inositol 3 phosphate kinase (PI3K) activation (1). Upon PI3K activation, membrane inositols are metabolized by PI3K into phosphatidyl inositol 3 phosphate (PI3P) and phosphatidyl inositol 2 phosphate (PI2P) (1). PI2P and PI3P metabolites bind the pleckstrin homology (PH) domain of Akt, recruiting Akt to the plasma membrane, where Akt is phosphorylated on Thr (308) (activation domain) and Ser (473) (hydrophobic domain) by PDK1 (phosphoinositide-dependent kinase) and other kinases (1).

Akt is expressed at baseline and/or induced or activated during immune cell stimulation, playing crucial roles in both innate and adaptive immune cells. In lymphocytes, TCR activation, cytokine, or chemokine signaling result in PI3K activation and therefore, Akt activation (1). Serial phosphorylation on Akt’s Thr (308) and Ser (473) residues is observed, resulting in kinase activity (1). While activated Akt remains close to the plasma membrane in several cell types, in lymphocytes active Akt is only transiently present at the plasma membrane. Instead, lymphocyte Akt activation results in quick plasma membrane release and translocation to the cytoplasm and nucleus (1). Consequences of Akt activation include mTOR activation (promoting protein synthesis), GSK-3 activation (promoting NFAT pathway and modulating metabolism), and forkhead transcription factor activation (regulating cell cycle and apoptosis/survival) (3). Akt is negatively regulated by phosphatases, particularly PTEN (which is deficient in Jurkat T cells resulting in constitutive Akt activity) and SHIP (4).

## Role of Akt in innate immune responses

### Akt in macrophages

Macrophages are innate immune cells that orchestrate adaptive immune responses by presenting antigens to lymphocytes and by releasing inflammatory mediators such as cytokines and chemokines (5, 6). Several reports have described Akt as an essential molecule that controls maturation, differentiation, and survival of myeloid cells *via* activation/repression of signaling pathways and transcription factors (7–9). For instance, phosphorylation of Akt regulates TLR expression, NF-κB activation, and cytokine production (7).

**Figure 1:**
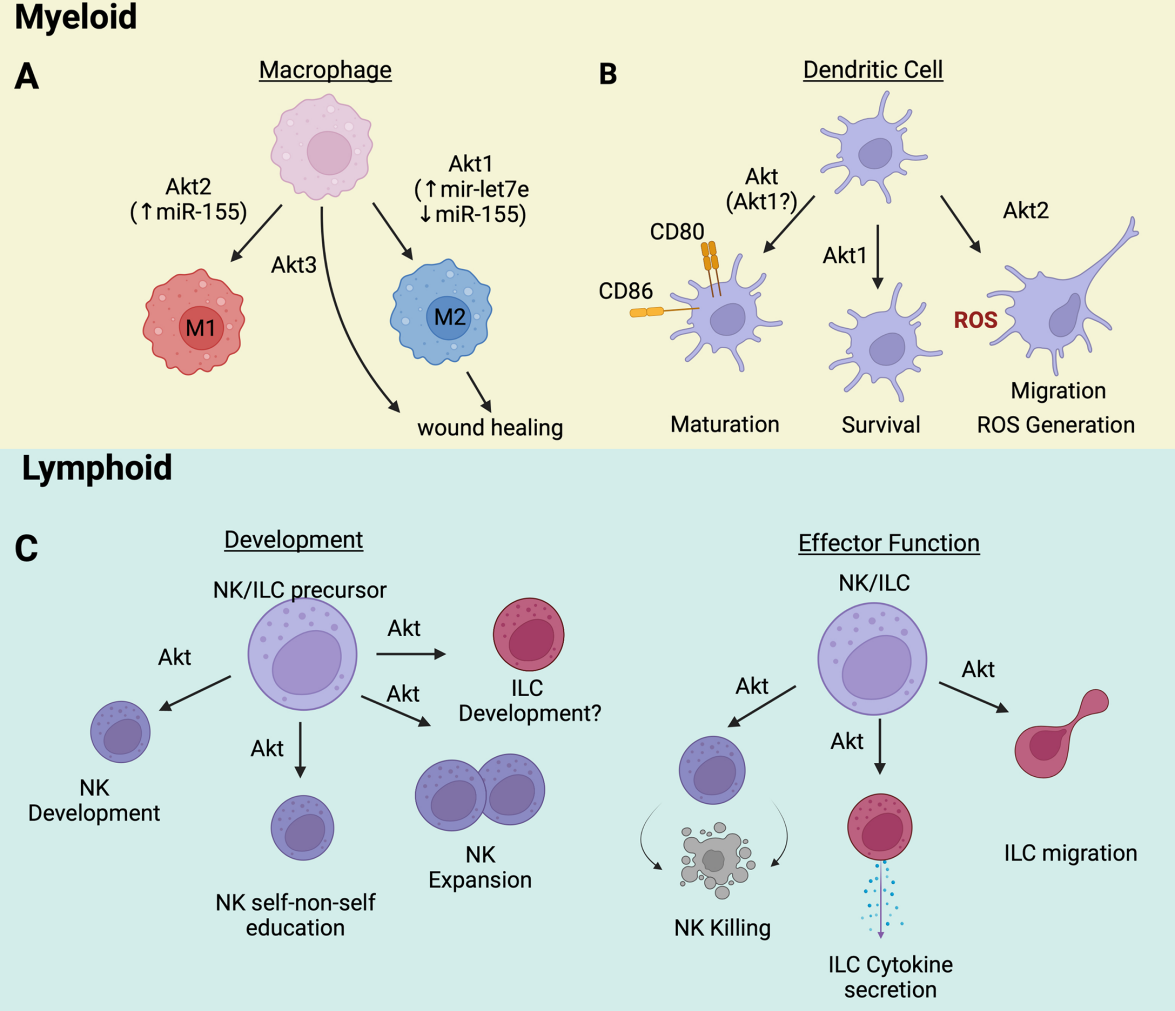
**(A)** Akt1/Akt2/Akt3 are directly involved in macrophage polarization and functions. **(B)** The maturation, survival, and function of dendritic cells rely on Akt activation. **(C)** Innate lymphoid cells development and effector functions are controlled by Akt signaling pathway. Wherever known, the targets and/or transducers of Akt’s effects are indicated in parenthesis. Image created with BioRender.com.

Macrophages exhibit significant plasticity, modulating their phenotype in response to environmental signals (10). This plasticity gives rise to significant macrophage diversity *in vivo*, the diversity reflecting the nature of the macrophage microenvironment. Much of the work on Akt in macrophages focuses on the *in vitro*-generated extreme phenotypes M1 and M2. M1 macrophages are proinflammatory while M2 macrophages display anti-inflammatory and wound-healing activities (11). Several lines of evidence point to Akt leading to M2 polarization, since deficiency or Akt signaling inhibition, abrogates upregulation of genes associated with the M2 phenotype (8, 12). Furthermore, it has been reported that TGF-β or IL-10-induced M2 polarization is dependent on PI3K/Akt signaling (8, 13, 14). The effects of Akt on macrophage polarization appear to be Akt isoform-specific^5,12^. Experiments with macrophages deficient in Akt1 or Akt2 revealed that whereas the ablation of Akt1 gives rise to M1 macrophages, the ablation of Akt2 gives rise to M2 macrophages (15). Thioglycolate-elicited macrophages from Akt1-deficient mice are hyper-responsive to LPS, producing high levels of inflammatory molecules such as TNF-α, IL-6, IL-17, IP-10, and iNOS (15, 16). The increased responsiveness and impaired tolerance of these macrophages may be mediated by the downregulation of miRNA let-7e, which represses TLR4, and by the upregulation of miR-155, which targets the negative regulator of macrophage activation SOCS1 (16). The opposite was observed in Akt2-deficient peritoneal macrophages. These cells were hyporesponsive to LPS. However, when they were treated with IL-4, which promotes M2 macrophage polarization, they produced high levels of Arginase 1 (Arg1) and IL-10. These effects were mechanistically linked to the downregulation of the pro-inflammatory miRNA miR-155 and the upregulation of its target C/EBPβ, which promotes the expression of Arg1 (15) (**Figure 1A**). As expected from these observations, Akt2^-/-^ and WT mice transplanted with Akt2^-/-^ macrophages were resistant to dextran sulfate sodium (DSS)-induced colitis (15).

IL-4-induced M2 macrophage polarization can be modulated by TGFβ. As a result, TGFβRII-deficient BMDMs, derived from vav-Cre/TGFβRIIfl/fl mice, are defective in their ability to undergo M2 polarization following IL-4 stimulation. Importantly, the M2 polarization defect of these macrophages is Stat6 and Smad3-independent but correlated with a reduction in Akt phosphorylation. However, the possibility that the loss of TGFβ signaling affects unequally the activity of Akt isoforms was not addressed in these experiments (14).

A study focusing on the IFN response in macrophages, also showed that Akt1 is selectively required for the induction of IFNβ by TLR3 activation signals. These signals activate Akt1, which phosphorylates β-Catenin at Ser522, and the phosphorylated β-Catenin induces the expression of IFN-β (17). Another study also showed that murine and human Akt1/AKT1 contribute to the cellular response to type I INFs (IFN-α and IFN-β). Specifically, AKT1 phosphorylates the transcriptional repressor EMSY at Ser209 and the phosphorylation of EMSY at this site relieves EMSY-mediated repression of IFN-regulated genes during viral infection (18).

The role of Akt3 in macrophage physiology is less well understood. Nonetheless, some evidence points to Akt3 exerting pro-regenerative functions independent of macrophage polarization. For instance, Gu et al. reported that delayed wound healing is associated with decreased Akt3 expression in M2 macrophages and decreased collagen production and skin fibroblast proliferation (**Figure 1A**). Although Akt3 expression in M2 macrophages was selectively reduced during delayed wound healing, macrophage polarization remained intact. Interestingly, downregulation of human AKT3 in M2 macrophages derived from the monocytic cell line THP1 also resulted in the downregulation of collagen expression and the inhibition of proliferation of cocultured human skin fibroblasts. Moreover, Akt3 knockout mice exhibited wound healing defects, and the cause of these defects was shown to be the loss of Akt3 in M2 macrophages. Overall, these data indicate that Akt3 selectively regulates the ability of M2 macrophages to promote tissue remodeling during wound healing by modulating the effects of macrophages on the function of skin fibroblasts, *via* a process that is independent of macrophage polarization (19).

### Akt in dendritic cells

Dendritic cells (DCs) are highly efficient professional antigen-presenting cells that promote the activation and differentiation of naïve T cells. Similar to macrophages, DCs have heterogeneous effector functions that are influenced by the DC microenvironment (20, 21). Thus, microenvironmental Toll-like receptor, TNF-α, IL-6, or IL-1β signals induce DC maturation (22). Mature DCs take up antigens and provide co-stimulation *via* CD40, CD80, and CD86 up-regulation (23). Recent studies provided evidence that the Akt signaling pathway is a required contributor to the maturation of bone marrow-derived DCs (BMDCs) (24). According to these studies, the maturation of BMDCs depends on the activation of PI3K/Akt signaling, and the process of PI3K/Akt activation is negatively regulated by B-cell adaptor for PI3K (BCAP). The inhibitory role of BCAP in this process was confirmed by experiments showing that LPS-stimulated BCAP-deficient DCs upregulate both the expression of CD80 and CD86 and the secretion of TNF-α, IL-6, IL-12, and MCP-1 (24).

The contribution of specific Akt isoforms to DC biology has also been explored (**Figure 1B**). DCs express only Akt1 and Akt2 (25). Akt1, the predominant isoform in BMDCs, is required for BMDC survival. Genetic ablation or pharmacological inhibition of Akt1 results in reduced expression of BMDC activation and maturation markers (9), suggesting that BMDC maturation depends on the activity of Akt1 (9). Akt2 appears to regulate DC effector functions such as chemotaxis and ROS generation. Lack of Akt2 impairs DC migration toward the chemo-attractants plasmin and CCL2 (25, 26). Importantly, plasmin also induces Akt2, but not Akt1, establishing a positive feedback loop between the chemo-attractant and the migration control molecule Akt2. DCs, like macrophages, kill pathogens *via* reactive oxygen species (ROS), which are induced by a process that requires Na^+^/H^+^ exchanger activity. In Akt2^-/-^ BMDCs the activity of the Na^+^/H^+^ exchanger is impaired, and the cytosolic pH is low. As a result, the cells have a defect in ROS production (26). Taken together, these observations show that Akt isoforms differentially promote the development and function of DCs.

### Akt in innate lymphoid cells

Akt also regulates the development and effector function of innate lymphoid cells (ILCs) (**Figure 1C**). ILCs are a family of immune cells that mirror the cytokine or cytotoxic activities of T cells but lack antigen-specific receptors. ILCs are classified in group 1, corresponding to natural killer cells (NK) cells and ILC1, group 2 (ILC2), and group 3 (ILC3). NK cells kill infected/tumor cells while ILCs initiate and modulate immune responses by producing cytokines normally associated with Th1 (ILC1), Th2 (ILC2), or Th17 (ILC3) cells.

NK cells generally mature in the bone marrow from common lymphoid progenitors (CLPs). An important step toward the NK cell fate is the expression of CD122, a receptor for IL-15. CD122 signals transduced by JAK kinases activate the PI3K/Akt pathway. Akt activation *via* Thr (308) and Ser (473) phosphorylation by PDK1 and mTORC2 respectively, promotes NK cell development (27, 28) as evidenced by the fact that this process is impaired when upstream (PI3K) or downstream (mTOR) Akt signaling is inhibited (29, 30). Akt also contributes to the “education” of NK cells, endowing them with the ability to distinguish self from non-self. Recently, it was shown that the Akt/mTORC2 signaling pathway, which is required for full Akt activation, is more active in “educated” NK cells and its activity correlates positively with the reactivity of NK cells toward non-self targets (31). Akt activation in NK cells can be facilitated by the upregulation of miR-155 and miR-21 and by the downregulation of the targets of these microRNAs, PTEN, PDCD4, and SHIP1. Activation of AKT by this mechanism stimulates NK cell expansion and contributes to NK cell lymphomagenesis (32). Less is known about the role of Akt in ILC development. However, ILCs, like NK cells, are derived from CLPs in the bone marrow and they share developmental stages with NK cells. Therefore, it would not be surprising if Akt is also a driver of ILC development.

Akt modulates the effector functions of both NK cells and ILCs. Even though they do not have antigen-specific receptors, NK cells kill MHC class I-deficient virus-infected or tumor cells. NK cell effector function depends on PI3K/Akt signaling and can be augmented by priming with cytokines such as IL-15 (33–37) or with factors that activate CRAC channels (38). Importantly, the priming also depends on the activity of Akt (34, 39, 40) and represents a potential strategy that can be employed to enhance the effectiveness of NK cell killing in cancer immunotherapy. In agreement with these observations, PTEN inhibits the cytolytic activity of NK cells by suppressing PI3K/Akt signaling (41).

ILC function also depends on the activity of the PI3K/AKT pathway. Recent studies have indeed shown that Akt activation by IL-33, IL-2, IL-7, and TSLP promotes the viability and proliferation of ILC2s and enhances allergic inflammatory responses *via* a process that is negatively regulated by NRF2 (42). ILC2 function is also promoted by Leptin-induced Akt activation in culture, in animal models, and in patients with allergic rhinitis (43). Finally, Akt activation contributes to ILC2 chemotaxis in IL-23 and CXCL16-induced allergic inflammation in a mouse model and in culture (44). In agreement with these observations, activation of the Leukocyte-associated immunoglobulin-like receptor (LAIR1), an ILC2 immune checkpoint inhibitory receptor, by its physiological ligand C1q in a humanized animal model inhibited Akt activation and protected the animals from allergic inflammation (45).

In other studies, Akt activation by signals induced by the bacterial metabolite receptor Ffar2, stimulates the proliferation of colonic ILC3s and the secretion of IL-22, modulating the host defense against intestinal pathogens and promoting intestinal homeostasis. Stimulation of the ILC3 receptor GPR34 by lysophosphatidylserine released from dying neutrophils recruited to the site of intestinal epithelial damage also promotes the secretion of IL-22 and contributes to the repair of the damage *via* a process that depends on Akt activation (46). Overall, these studies show that the PI3K/Akt pathway promotes the development and activity of NK cells and ILCs and that targeting this pathway could be used to regulate innate lymphoid cell effector function.

## Akt in adaptive immunity

### Akt in αβ T cells

#### Akt in CD4 Th lymphocytes

Productive T cell activation requires two signals, one provided by antigen-specific TCR engagement, and another provided by ligand binding to costimulatory receptors such as CD28. Costimulatory CD28 signals result in production of survival and growth-promoting cytokines such as IL-2 and are also essential for Th1 and Th2 cytokine production. Some of the signals initiated by CD28 co-stimulation are transduced by Akt. Thus, activation of Akt by CD28 co-stimulation signals is required for IL-2 and IFN-γ induction and constitutively active Myr-Akt is sufficient to induce these cytokines even if CD28 signaling is blocked. In contrast, CD28-dependent production of Th2 cytokines IL-4 and IL-5 is promoted by an Akt-independent mechanism.

Akt also regulates the generation and function of Tregs *via* complex and in some cases, not well-defined mechanisms. There are two main types of Tregs, the natural Tregs (nTregs), which develop in the thymus and migrate to the periphery, and the induced Tregs (iTregs), which develop in the periphery from naïve CD4+ T cells, following suboptimal antigen stimulation in the presence of various cytokines, primarily TGFβ (47). The Treg transcriptional program for the development of both nTregs and iTregs is induced by TCR stimulation and co-stimulatory signals that are transduced *via* the PI3K/Akt pathway. However, whereas the program for the development of iTregs also depends on cytokine signals such as TGFβ, the nTreg program is TGFβ-independent.

Several pieces of evidence suggest that strong activation of the PI3K/Akt/mTOR pathway inhibits the development of both nTregs and iTregs. Thus, p110δ kinase-inactive knockin mice have a higher-than-normal percentage of nTregs in the thymus, probably because of an attenuation of PI3K/Akt signaling during positive selection (48). In agreement with this observation, the number of nTregs in the thymus in Foxo1/Foxo3a double knockout mice is greatly diminished. The FOXO transcription factors bind a consensus sequence in the Foxp3 promoter and activate Foxp3 gene expression. Activated Akt phosphorylates these factors and interferes with their nuclear translocation, and as a result, it inhibits the expression of the Treg specification factor, Foxp3. Moreover, deletion of Sin1, a component of the mTORC2 complex, results in the attenuation of Akt activity and an increase in the number of nTregs in the thymus (49). Finally, both nTregs and iTregs express higher levels of PHLPP1 and PHLPP2, two phosphatases, which dephosphorylate the Se473 Akt phosphorylation site and attenuate its activity (50). The inhibition of iTreg development by strong PI3K/Akt signals is also supported by multiple publications (51–54).

The PI3/Akt pathway has also been reported to regulate the function of Tregs and the sensitivity of conventional CD4+ and CD8+ T cells to the immunosuppressive activity of Tregs. Thus, it has been shown that Sphingosine-1-phosphate (S1P) activates the PI3K/Akt/mTOR pathway, which attenuates not only the differentiation of thymic Treg precursors, but also the functional activity of nTregs, giving rise to a proinflammatory phenotype (55). In other studies, it was shown that mouse Tregs express high levels of FOXO1 and they display reduced TCR-induced Akt activation, a prerequisite for the stable localization of FOXO1 in the nucleus. The significance of this observation was underscored by the finding that Treg-specific deletion of FOXO1 gives rise to a fatal inflammatory syndrome in mice (56). The effects of FOXO1 phosphorylation by Akt in Tregs were addressed directly with the Treg-specific expression of an Akt-insensitive FOXO1 mutant. This experiment confirmed that Akt-mediated FOXO1 phosphorylation results in the downregulation of lymphoid organ homing molecules and promotes the migration of the activated Tregs from secondary lymphoid organs to non-lymphoid tissues (57). Akt activity may also attenuate the sensitivity of conventional T cells to the immunosuppressive activity of Tregs. This was observed in transgenic mice expressing constitutively active MyrAkt1 in T cells (58), as well as in TRAF6 knockout mice. TRAF6 deletion results in Akt activation, which renders conventional T cells resistant to Treg suppression, giving rise to a proinflammatory phenotype (59). Whereas these studies suggest that Akt inhibits Treg function and the response of the target cells to the Treg immunosuppressive activity, other studies suggest the opposite. Thus, mTORC1, a downstream target of Akt, has been identified as a pivotal positive determinant of Treg function. Tregs express high steady-state levels of mTORC1 activity compared to naïve conventional T cells and Raptor deletion results in an early onset proinflammatory syndrome in mice. Finally, mTORC1 is required for the induction of CTLA4, ICOS, and several enzymes involved in lipid metabolism in Tregs, but it does not affect the expression of Foxp3 (60).

**Figure 2:**
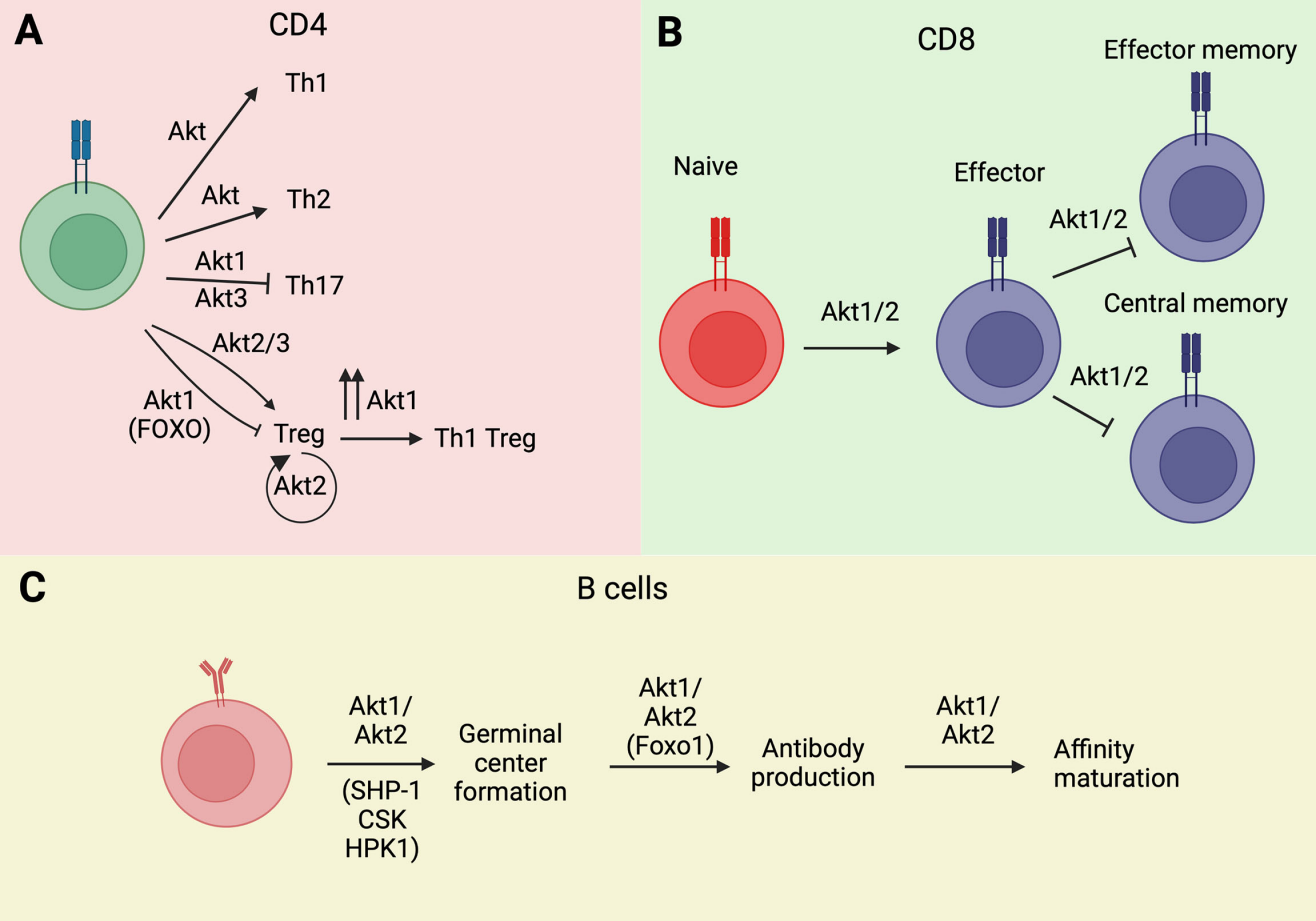
Akt isoforms regulate lymphocyte ontogeny and differentiation. **(A)** Akt1 and Akt3 isoforms modulate Th differentiation of CD4 Th cells. **(B)** Similarly, Akt1 isoform is implicated in memory phenotype acquisition in T CD8^+^ cells. **(C)** The activation of Akt1/Akt2 isoforms is essential for B cell ontogeny. Wherever known, the targets and/or transducers of Akt’s effects are indicated in parenthesis. Image created with BioRender.com.

Some of the effects of Akt on the generation and function of Tregs are isoform-specific (**Figure 2A**). Moreover, since Tregs profoundly affect the susceptibility to and the biology of autoimmune diseases and cancer, the Akt isoform specificity on the generation and function of Tregs has strong translational implications. Akt2 selectively promotes the proliferation of Foxp3+ Tregs and its ablation inhibits Treg development. Akt1 has the opposite effect. As a result, its inhibition with an Akt1-specific inhibitor inhibits MOG-induced autoimmune encephalomyelitis in mice (61). Moreover, strong human AKT1 activation by CCL3 results in K48-linked ubiquitination and degradation of human FOXP3 and exacerbates psoriasis (62). The effect of Akt3 on Treg development and function is similar to the effect of Akt2. Therefore, ablation of Akt3 enhances the sensitivity of mice to EAE. Moreover, an activating Akt3 mutation (D219V) is neuroprotective, and the neuroprotection is due to the enhanced Akt3 activity in T cells but not in neurons (63). Importantly the enhanced sensitivity of Akt3-/- mice to EAE, was not only due to Treg deficiency but also to the decreased sensitivity of their Th1 and Th17 cells to Treg-mediated immune suppression (64) (**Figure 2A**).

Tregs exhibit significant plasticity and the regulation of this plasticity depends on Akt. TCR stimulation of Tregs, combined with IL-12 stimulation, induces the expression of IFNγ and the IL-7 receptor (CD127), which is not normally expressed in Tregs. The reprogrammed cells are known as Th1-Tregs, which secrete IFNγ, and even though they continue to express human FOXP3, they do not suppress the activity of conventional T cells. Importantly, Akt1 is required for the Treg to Th1-Treg reprogramming. In contrast, not only Akt3 is not required, but its silencing is sufficient to induce the phenotype. In agreement with these observations, the ablation of Akt1 protects, while the ablation of Akt3 promotes the development of experimental autoimmune encephalomyelitis (EAE) (65). A more recent study identified an Akt isoform-specific effect on Treg reprogramming in hyperlipidemic mice. This study showed that hyperlipidemia selectively activates Akt2 in Tregs and that Akt2 activation promotes glycolysis and the secretion of proinflammatory cytokines. Therefore, selective activation of Akt2 by hyperlipidemia promotes inflammation by functionally reprogramming the Tregs (66).

As mentioned above, iTregs are induced by stimulation of naïve CD4+ T cells with TCR/costimulatory molecule signals, combined with cytokine (primarily TGFβ) signals. Another cell lineage that is derived from the same progenitor cells and is induced by overlapping signals is the Th17 lineage. The decision between Treg and Th17 cell differentiation depends on the microenvironment, with an inflammatory microenvironment characterized by the abundance of proinflammatory cytokines, such as IL-6 or IL-21, favoring differentiation toward the Th17 lineage. Mechanistically, the signals that promote Th17 cell differentiation activate STAT3, which induces (ROR)γt, the Th17 cell specification factor. This is an important decision, because whereas Tregs have anti-inflammatory activity, Th17 cells secrete the pro-inflammatory cytokines IL-17, IL-22, and IL-23, promoting inflammation and autoimmunity. Evidence to-date indicates that this decision is regulated at least in part by CK2, perhaps *via* Akt (67, 68). Increased Th17 differentiation in Akt3 deficient mice supports a negative regulatory role for the Akt3 isoform in Th17 differentiation (63).

#### Akt in CD8 Tc lymphocytes

CD8 T cell responses are essential for anti-viral and anti-cancer immunity. Naïve CD8 T cells encountering cognate viral or cancer antigens undergo activation, characterized by proliferation and the acquisition of effector functions. Some of the activated cells become memory CD8 T cells that live long and respond rapidly, upon re-exposure to the same antigen. These can be either central memory (T_CM_) or effector memory (T_EM_) CD8 cells. T_CM_ cells reside in lymph nodes and exhibit the highest proliferative and effector functions upon re-exposure to the antigen. Terminally differentiated effector cells ultimately become exhausted. Therefore, the acquisition of the memory phenotype is considered essential for viral and cancer immunity.

Abu Eid et al. found that Akt isoforms Akt1 and Akt2 promote the terminal differentiation of CD8 T cells into effector cells and interfere with the acquisition of the T_CM_ and T_EM_ phenotypes (69) (**Figure 2B**). As a result, inhibition or loss of Akt1 and Akt2, but not Akt3, increased T_CM_ cell numbers and proliferative responses (69). Importantly, multiple restimulations of Akt1 and Akt2 knockout CD8 T cells led to an increase rather than a decrease in cytotoxic responses, as measured by granzyme B expression and IFN-γ/TNF-α cytotoxic cytokine production (69). These results suggest that inhibition of Akt1 and Akt2 may be effective at maintaining cytotoxic anti-cancer immune responses and that it may be therapeutically beneficial in cancer (69). Importantly, the PI3K isoform responsible for the activation of Akt1 and Akt2 and the promotion of terminal differentiation of CD8 T cells was identified as the PI3Kδ isoform (70). As a result, PI3δ inhibition in a model of tumor vaccination, enhanced vaccine effectiveness and tumor regression (70).

#### Akt in αβ T cell development

T cells develop and achieve tolerance to self-antigens in the thymus. Central to the development of T lymphocytes are the antigen-specific receptor rearrangements, which trigger the processes of positive and negative selection. In T cells, a successful rearrangement of the beta chain of the T cell receptor (TcR) and its assembly with pre-T alpha provides signals for survival and proliferation and is known as beta selection. This occurs during the CD4^-^CD8^-^ double negative 3 (DN3) stage and it is required for the transition to the DN4 stage, which precedes the CD4^+^CD8^+^ double positive (DP) stage of thymocyte development (71).

Analyses of Akt isoform expression during T cell development in the thymus revealed that the major expressed isoforms during the DN stage are Akt1 and Akt2 (72). Akt3 was expressed at much lower levels (72, 73), but its expression increased in the DP and SP stage, as well as in mature T cells in secondary lymphoid organs (72). This expression pattern suggested that Akt1 and Akt2 are the major isoforms involved in the regulation of thymocyte development. This hypothesis was ultimately confirmed by experiments showing that the assembly and activation of the preTCR (TCRβ/preTα) during the DN3 stage promotes the transition of DN3 cells to the DN4 stage *via* a process that depends on the activation of Akt1 and Akt2 by preTCR-initiated signals. Akt1 and Akt2 were also shown to promote the survival and proliferation of DN4 cells, prior to their transition to the immature CD8 Single positive (ISP) stage. Finally, they were shown to promote the survival of DP thymocytes undergoing thymocyte selection (73). Single Akt isoform deletion and combined deletion of Akt1 and Akt3, or Akt2 and Akt3, had only minor effects on thymocyte development, suggesting that Akt1 and Akt2 can complement each other in thymocyte differentiation and that Akt3 is not a major contributor to the process of thymocyte development (72, 73). Additional support to the conclusions drawn from the preceding data was provided by experiments showing that a constitutively active (myristoylated) Akt1 promotes thymocyte development with enhancement of the transition from the DN3 to the DN4 stage of thymocyte development (72). Mechanistically, it appears that the effects of Akt1 and Akt2 on thymocyte development may be secondary to their effects on thymocyte metabolism. The loss of Akt1 and Akt2 indeed reduced glucose uptake and blocked proliferation at the DN4 stage. Moreover, the complete loss of Akt signaling in triple Akt1/2/3 KO T cells resulted in reduced DN thymocyte survival (72, 73).

The process of β-selection is also regulated by Notch, and the phenotype of Notch deletion can be rescued by constitutively active Akt1. Overall, studies of Akt isoforms during thymocyte development show that Akt1 and Akt2 play crucial roles during T cell development in the thymus.

### Akt in γδ T cells

Mammalian ɣδ T cells express T cell receptors composed of rearranged γ and δ chains. They differ from classical αβ T cells in that whereas the αβ T cells adopt effector functions only after antigen stimulation, ɣδ T cells commit to the effector fate during their development in the thymus (74). As a result, similar to cells of the innate immune system, they respond rapidly to infections by viruses and bacterial pathogens, and they exhibit strong, MHC-unrestricted cytotoxicity against tumor cells (75).

ɣδ T cells develop in the thymus and they are derived from the same thymocyte progenitor cells as the αβ thymocytes (74). The two thymocyte lineages diverge during the early DN stage of thymocyte differentiation and their divergence is triggered by the rearrangement of different chains of the T cell receptor. Akt is playing a well-established critical role in the development of αβ T cells (see αβ cell development section), but its role in the development of ɣδ T cells is less well understood. Recent studies addressing this question revealed that Akt plays a complex but elegant role in the development of a ɣδ T cell subset, the ɣδ^17^ cells. There are two main subsets of ɣδ T cells, the ɣδ^IFN^ cells, which secrete ɣ-IFN, and the ɣδ^17^ cells, which secrete IL-17A. The ɣδ^IFN^ cells are induced by strong ɣδ TCR signals, which activate the ERK pathway, while the ɣδ^17^ cells are induced by weaker signals, which activate the receptor proximal tyrosine kinase Syk and the PI3K/Akt pathway, promoting the expression of key transcriptional regulators of the IL-17 program, such as *Rorc* and *Maf*. Importantly, the required level of Syk/PI3K/AKT activity is constrained within relatively narrow limits, as both the strong activation and the inhibition of the pathway inhibit ɣδ^17^ T cell development. Also, whereas strong activation of the pathway shifts the differentiation toward the ɣδ^IFN^ T cell subset, inhibition of the pathway results in the development of a novel CD8+ Ly6a+ innate-like ɣδ T cell (76). Both Syk and its downstream targets PI3K and Akt are critical components of the pathway. Thus, Zap70, another receptor proximal tyrosine kinase failed to substitute for the loss of Syk, and the ablation of RhoH, an adaptor for Syk, ameliorated skin inflammation, in a ɣδ^17^-dependent skin inflammation model in mice. Moreover, mice deficient in PI3K/AKT signaling exhibited complete loss of ɣδ^17^ cells, without any impairment in the development of ɣδ^IFN^ T cells (77).

AKT contributes not only to the regulation of ɣδ T cell development, but also to the functional regulation of these cells. First, it contributes to the activation of ɣδ T cells by natural ligands, such as metabolite phosphoantigens. Vɣ9Vδ2, the main human ɣδ T cell subset, responds to the phosphoantigen Isopentenyl-pyrophosphate (IPP), which is produced in mammalian cells by the mevalonate pathway. The PI3K/AKT/mTOR pathway inhibits the nuclear translocation of LXRα and the expression of the ATP binding cassette transporter A1 (ABCA1) and apoA-I, which synergistically promote the release IPP. Inhibition of the PI3K/AKT/mTOR pathway, therefore, increases the expression of these transporters, the release of IPP, and the activation of Vɣ9Vδ2 ɣδ T cells. Zoledronic acid (ZA), an inhibitor of farnesyl-pyrophosphate synthase (FPPS), an enzyme in the mevalonate pathway, activates ɣδ T cells, *via* this mechanism. Specifically, ZA increases the intra- and extra-cellular level of IPP, which triggers Vɣ9Vδ2 in ɣδ T cells, inhibiting Akt and enhancing further the release of IPP and ɣδ T cell activation (78).

Akt activated downstream of Casein Kinase-2 (CK2), inhibits apoptosis of ɣδ T cells. Normal human ɣδ T cells and ɣδ T-ALL cells express higher levels of CK2 than normal and leukemic αβ T cells. As a result, ɣδ T-ALL cells are more sensitive than αβ T-ALL cells to CK2 inhibition (79). Finally, Akt activation promotes the cytotoxic activity of ɣδ T cells. Earlier studies had shown that the cytotoxic activity of the main subset of ɣδ T cells (Vɣ9Vδ2) against leukemic cells tends to be blocked during tumor progression (80). However, the cytotoxic activity of Vδ1, another common ɣδ T cell subset, is not. The cytotoxic activity of these cells depends on the expression of the natural cytotoxicity receptors (NCRs) NKp30, NKp44, and NKp46 and on Granzyme B, which are induced by Akt-dependent ɣ chain cytokine (IL-2 and IL-15) signals (81).

#### Akt in B lymphocytes

Splenic B cells express all three Akt isoforms. Among them, Akt1 is the most highly expressed (82). B cells in secondary lymphoid organs such as the spleen and lymph nodes, encounter antigens drained from the surrounding tissues, and they become activated in response to the combination of antigen exposure and helper T cell-derived signals. Following activation, B cells proliferate and differentiate into antibody-producing plasma cells. B cells in secondary lymphoid organs (lymph nodes and spleen), responding to T cell-dependent antigens, form germinal centers (GCs), where they proliferate, undergo immunoglobulin class switching, and secrete antibodies with increasing affinity to antigen, *via* the process of affinity maturation. Affinity maturation requires iterative somatic hypermutation of selected clones and subsequent selection of clones producing antibodies with increasing affinity to the antigen. GC B cells integrate B cell receptor (BCR) signals with helper T cell-derived signals to achieve affinity maturation and B cell differentiation (83).

The PI3K/Akt pathway plays an important role in GC B cell selection. Of the three Akt isoforms, Akt1 and Akt2 are essential for the formation of GCs and the processes of affinity maturation and antibody production (82) (**Figure 2C**). Akt1/2 double deficiency resulted in impaired glucose uptake and oxidative phosphorylation in B cells exposed to T cell-dependent antigens, and this led to reduced B cell proliferation and survival and restricted GC development (82). Class switch recombination and IRF4-dependent plasma cell differentiation depend on the Akt phosphorylation target Foxo1 (82). Mutations in one of the Foxo1 residues phosphorylated by Akt, keeps Foxo1 in its active nuclear form, promoting class switch recombination *via* the induction of the activation-induced cytidine deaminase (AICD) (82). This mutation has also been observed in GC Large B cell lymphomas.

It has been proposed that the role of Akt may be less of a positive/negative switch and more of a “tuner” of antigen-induced signals (84). Thus, BCR signals are attenuated in GC B cells relative to naïve B cells (85–87) and Akt promotes BCR signal attenuation by phosphorylation-induced activation of three negative regulators of BCR signaling, namely, SHP-1, CSK and HPK1 (4). This is not observed in naïve B cells and is therefore a GC-restricted Akt-dependent B cell mechanism, designed to maintain the diversity of antigen-specific receptors and to ensure optimal affinity maturation (84).

## Concluding remarks

This review summarizes the role of the three Akt isoforms in immune cell development, and in the regulation of the immune response (**Table 1**). The discovery that Akt is a downstream target of the PI3 Kinase (94), which followed the initial discovery of Akt in 1991 (95), placed the kinase encoded by *Akt* at the center of a major signaling pathway in all cells. The research effort that followed, produced almost 110,000 publications, addressing the role of Akt in all aspects of cell and organismal biology. Despite the enormous progress however, important questions remain.

**Table 1:** Akt isoform animal models provide insights into their immunological roles.

Akt isoform	Mouse model		Role of Akt in the immune system	Refs
**Akt1**	Myristoylated Akt	myrAkt CD28^-/-^	• Enhanced Akt signaling promotes iTregs by replacing CD28 signaling requirements. • Akt1 signaling impairs TGF-β1/IL-6-mediated differentiation of naïve CD4^+^ T cells. • Akt1 signals decrease EAE severity.	(9, 15, 16, 26, 58, 61, 69, 88–90)
		CD2 myrAkt	• Constitutive Akt1 signaling contributes to proliferation, activation, and selection of thymocytes by promoting signaling downstream of TCR. • myrAkt mice accumulate memory-phenotype CD4^+^ T cells, B cells, and develop both tumors and autoimmunity. • Akt overexpression improves survival during sepsis.	
	Akt1^-/-^		• Akt1 suppresses the proliferation of thymus-derived Treg cells and promotes Th1/Th17 responses. • Akt1 is required for DC survival, maturation, and activation. • Absence of Akt1 promotes M1 polarization of macrophages. • Akt1 controls antigen-specific CD8^+^ T cells differentiation.	
**Akt2**	Akt2^-/-^		• Akt-2 promotes tTreg proliferation and suppresses Th1/Th17 responses. As consequence, Akt2 deficient mice are more susceptible to EAE.	(15, 61, 63, 64, 69, 82, 91)
			• Akt2 regulates interferon type 1 responses. • Akt2^-/-^ mice have reduced mortality in response to viral infection but aggravates the severity of SLE. • Deficiency of Akt2 promotes anti-inflammatory macrophages. • Akt2 regulates ROS production and migration of DC • Akt2 promotes differentiation of CD8^+^ T cells.	
	Akt1/2^-/-^ Cγ1^Cre^ Akt1^f/f^ Akt2^-/-^		• Akt1/2 controls function and auto-renewal capacity of HSC by regulating cell cycle and intracellular ROS. • Absence of Akt1/2 inhibits proliferation of DN4 (CD44^-^CD25^-^) cells and survival of DP thymocytes. • Akt1/2 signaling is necessary for formation of germinal center, affinity maturation and antibody production	
**Akt3**	Akt3^Nmf350^ (enhanced activity) CD4-Cre^+^ Akt3^fl/fl^ Syn1-Cre^+^ Akt3^fl/fl^ Akt3-/-		• Akt3 signaling in T-cells and not neurons maintain CNS integrity during inflammatory demyelinating diseases. • Absence of Akt3 in spinal cords favors demyelination, high levels of inflammatory cytokines, and decrease of regulatory T cells.	(19, 72, 92, 93)
	Akt1/2/3^-/-^		• Akt1/2/3^-/-^ regulates thymocyte selection by inhibiting proliferation and survival of DN cells.
	Akt3^-/-^		• AKT3 inhibits formation of foam cells and actin-dependent micropinocytosis. • Absence of Akt3^-/-^ in M2 macrophages impairs tissue remodeling and wound healing.

Given the central role of Akt in cell signaling, perturbations of the Akt pathway have been linked to human disease, including cancer and autoimmunity. Based on this, Akt was explored as an important target for the treatment on human disease. Surprisingly, the results to-date have been rather disappointing (96, 97). One of the reasons is the high toxicity of Akt inhibitors, which stems from the fact that Akt has a central role in cell signaling in all cells. Another reason is that Akt exists in three isoforms, which have unique in in some cases opposing functions. For instance, Akt1 and Akt2 have opposing roles in macrophage polarization (8, 15, 16) and in Treg development (61–64). Further understanding of the unique roles of the three Akt isoforms in all the cells of the immune system, as well as in the regulation of immune processes, such as Th1 or Th2 cell differentiation, and in the regulation of mucosal immunity, along with the development of Akt isoform-specific inhibitors (98, 99). may provide actional information for the successful targeting of Akt in human disease. We should add that given the fact that Akt is an important regulator of cellular metabolism (31686003) and that metabolism is known to drive immune cell function, understanding the role of Akt isoforms in the metabolism of all cells of the immune system may provide additional actionable information for the targeting of Akt-driven metabolic pathways in the immune system. Developing novel Akt isoform-based strategies to target various immune cell types may lead to successful immunotherapeutic approaches for the treatment of both cancer and autoimmune diseases in humans.

## Author contributions

MG-d-A wrote most sections and edited the manuscript, ZP-Q wrote the myeloid innate immune cells section and edited the manuscript and PT wrote the gamma delta T cell sections, heavily edited the Treg section and overall edited the manuscript. All authors contributed to the article and approved the submitted version.

## Conflict of interest

MG-d-A is an inventor on a PRMT5 inhibitor patent.

The remaining authors declare that the research was conducted in the absence of any commercial or financial relationships that could be construed as a potential conflict of interest.

## Publisher’s note

All claims expressed in this article are solely those of the authors and do not necessarily represent those of their affiliated organizations, or those of the publisher, the editors and the reviewers. Any product that may be evaluated in this article, or claim that may be made by its manufacturer, is not guaranteed or endorsed by the publisher.
